# Plant MicroRNAs in Cross-Kingdom Regulation of Gene Expression

**DOI:** 10.3390/ijms19072007

**Published:** 2018-07-10

**Authors:** Wei Wang, Dan Liu, Xiaopei Zhang, Dongdong Chen, Yingying Cheng, Fafu Shen

**Affiliations:** State Key Laboratory of Crop Biology, College of Agronomy, Shandong Agricultural University, Tai’an 271018, China; weiwang@sdau.edu.cn (W.W.); zkybliudan@163.com (D.L.); xpzhang0108@163.com (X.Z.); 18264893878@163.com (D.C.); 17661212080@163.com (Y.C.)

**Keywords:** plant, microRNA, host, pathogens, cross-kingdom regulation

## Abstract

MicroRNAs (miRNAs) are a class of noncoding small RNAs, which play a crucial role in post-transcriptional gene regulation. Recently, various reports revealed that miRNAs could be transmitted between species to mediate cross-kingdom regulation by integrating into a specific target gene-mediated regulatory pathway to exert relevant biological functions. Some scholars and researchers have observed this as an attractive hypothesis that may provide a foundation for novel approaches in the diagnosis, prognosis, and treatment of disease. Meanwhile, others deem the mentioned results were obtained from a “false positive effect” of performed experiments. Here, we focus on several current studies concerning plant miRNA-mediated cross-kingdom regulation (from both fronts) and discuss the existing issues that need further consideration. We also discuss possible miRNA horizontal transfer mechanisms from one species to another and analyze the relationship between miRNA-mediated cross-kingdom regulation and coevolution during a long-term specific host–pathogen interaction.

## 1. Introduction

MicroRNAs (miRNAs), a class of single-stranded noncoding small RNAs (sRNAs) ranging in size from 20 to 24 nucleotides in mature sequence length, have been shown to be master modulators of gene expression by binding to the open reading frame (ORF) or untranslated region (UTR) of specific mRNAs, targeting them for cleavage or by directing translational inhibition at the mRNA level [1]. In the past few years, miRNAs have been shown to play crucial roles in various critical biological processes associated with cell growth and differentiation, cell proliferation, apoptosis and immune response, and the regulation of some important agronomic traits [2,3,4,5,6].

Growing evidence reveals that miRNAs target not only endogenous genes, but also exogenous genes. In 2009, researchers at Monsanto Company found that numerous endogenous plant miRNAs exhibited perfect complementarity to human genes as well as to those of other mammals [7]; this was followed, in 2011, by the research of Nanjing University Professor Chen-Yu Zhang’s team, which confirmed that plant-derived miR168a could be absorbed through the gastrointestinal tract into mammalian liver cells, where it inhibited the expression of the human/mouse low-density lipoprotein receptor adapter protein 1 (LDLRAP1), and consequently decreased LDL removal from mouse plasma [8]. These spectacular findings raised the possibility that exogenous miRNAs may cross species barriers and serve as signaling factors, regulating gene expression and physiological function, affecting or indicating the organism’s health, and further broadening the understanding of cross-kingdom communication at the same time. Moreover, these studies launched a wave of publications competing arguments and started fierce debate among scientists that continues to this day. This hypothesis, in which the cross-kingdom regulation is mediated by exogenous plant miRNAs, is still controversial and remains to be demonstrated consistently and unequivocally.

Here, we highlight several recent discoveries concerning cross-kingdom gene regulation mediated by plant miRNAs and analyze the existing issues that need further consideration. We also discuss possible miRNA transport mechanisms from one species to another and analyze the relationship between miRNA-mediated cross-kingdom regulation and coevolution during a long-term specific host–pathogen interaction.

## 2. Plant miRNAs Regulate Gene Expression in Animals

The ability of miRNAs to regulate gene expression across kingdom has already been shown for viruses and parasites [9,10], whereas research from the Monsanto Company [7], followed by the work of Zhang et al. [8], was the first to present evidence to demonstrate these intriguing properties for plant miRNAs. In the study carried out by Monsanto Company researchers, an estimate of sRNA abundance was provided through semiquantification of endogenous sRNAs in grains (soybean, corn, and rice), and the sequences of numerous endogenous plant miRNAs were found to be complementary to parts of the genomes and transcriptomes of humans and other animals [7]. This group considered that the fact that these endogenous plant miRNAs derived from staple food crops with a long history of safe consumption by humans and domesticated animals provided evidence that consumption of these mediators of RNA interference was safe, and supported the safety of this technology for use in biotechnology-derived crops [7]. Furthermore, the ground-breaking research of Zhang et al. in 2011 [8] has not been reproduced to date [11].

Although the proposal that plant miRNAs may function in a cross-kingdom manner was controversial, the report of Zhang et al. [8] motivated many scientists to investigate this issue in more depth. A recent finding by Andrew et al. showed that plant miR159 could be taken up from certain types of dietary sources into human sera in Western subjects at levels that were inversely correlated with breast cancer (BC) incidence and progression in patients [12]. This research group further identified a synthetic mimic of plant miR159, which was capable of inhibiting cell proliferation in an in vitro model by targeting *TCF7*, which encodes a Wingless-related integration site (Wnt) signaling-associated transcription factor, leading to a decrease in MYC transcription factor protein levels in BC cells. Moreover, oral administration of the miR159 mimic significantly suppressed the growth of xenograft breast tumors in mice using in vivo models [12]. The study confirmed that orally delivered plant miRNAs could be detected in human sera and revealed for the first time that a plant miRNA was able to influence cancer growth in mammals in vitro. Another recent publication, from Yu-Chen Liu and colleagues, supports the hypothesis of cross-kingdom regulation mediated by plant miRNAs because plant miRNA sequences were identified in human sera through analysis of publicly available plasma sRNA sequencing datasets [13]. Shortly afterwards, however, the most abundant plant miRNA identified, peu-miR2910, present at only 5369 copies, was removed from the miRBase database (http://www.mirbase.org/) due to being a fragment of a large-subunit ribosomal RNA [14]. In addition, a number of recent reports suggest that evidence of exogenous plant miRNAs in human samples obtained by sequencing methods is subject to artefactual bias [14,15,16].

Recently, Ke-Gan Zhu et al. reported a previously uncharacterized regulatory mechanism in honey bee caste development, which can be partially attributed to plant miRNAs, a heretofore overlooked component of larval food [17]. It is generally known that there are normally three social classes in a honey bee colony: drones, workers, and a single queen. The drones are haploid, short-lived male bees that develop from unfertilized eggs. The queen and workers both develop from fertilized eggs and are thus genetically identical, but differ in terms of morphology, physiology, and social function. The former is reproductive, has a larger body size, develops faster, and lives longer, while the latter are mostly sterile helpers which collect food and nourish the larvae, and they are characterized by traits different from those of the queens [18,19]. This queen–worker dimorphism is not a consequence of genetic differences, but is mediated socially by larval feeding: royal jelly stimulates the differentiation of larvae into queens, whereas beebread determines the workers’ fate. Royal jelly, a glandular secretion of nurse bees, is animal-derived, while beebread, a mixture of pollen and honey, is plant-derived.

Studies have revealed that some insects can ingest sRNAs, which can subsequently regulate the expression of insect genes, thus reshaping the phenotype of the insect [20,21]. Therefore, researchers have hypothesized that the different miRNA contents of larval food of different origins may have distinct impacts on honey bee development. Firstly, utilizing Illumina deep-sequencing technology, qRT-PCR assays, and northern blot analyses, Ke-Gan Zhu et al. analyzed the sRNA components in royal jelly, honey, beebread, and pollen, and observed that 16 plant miRNAs were at higher concentrations in beebread and pollen than in royal jelly, and also found that three plant miRNAs (miR156a, miR162a, and miR168a) were detectable in beebread and pollen, but not in royal jelly or honey [17]. To investigate the effects of these plant miRNAs on honey bee phenotype, researchers reared larvae on a laboratory diet with or without the addition of the 16 plant miRNAs present at higher concentrations in beebread and pollen, and observed that larvae which were fed a diet containing these plant miRNAs developed worker-bee-like characteristics [17]. Subsequently, bioinformatic analysis was performed to dissect the potential functions of the plant miRNAs in honey bee food, and 96 genes that may be involved in the regulation of the caste development process of honey bees were predicted to be targeted by the 16 plant miRNAs. The *Apis mellifera* L. target of rapamycin (TOR) (*amTOR*) gene, known to play a stimulatory role in caste development, was specifically selected to analyze the targeting relationship with the 16 plant miRNAs, using luciferase reporter assays and qRT-PCR analysis. Comparing the corresponding phenotypes of honey bee larvae which were reared on a diet to which either synthetic miR162a or scrambled RNA (which had no effect on any of the tested morphological characteristics) was added, the results showed that plant miR162a specifically recognized *amTOR* and downregulated its expression at the post-transcriptional level [17]. Thus, the study revealed that the development of larvae into workers rather than the queen was due, at least in part, to *amTOR* knockdown by plant miR162a present in beebread.

During honey bee caste development, one- and two-day-old worker larvae are fed secretions from nurse bees’ hypopharyngeal and mandibular glands. They are fed small amounts of pollen (obtained from beebread) after the third larval instar. Furthermore, larvae that turn into queens must be fed royal jelly only during the first three larval instars [22,23]. In addressing the problem of miRNA feeding method in the study of Ke-Gan Zhu et al. [17], in our view, pollen feeding, which takes place after the third larval instar, should not matter in terms of queen–worker destiny.

Generally, this discovery of so-called cross-kingdom regulation of gene expression indicates a potentially therapeutic role for plant miRNAs, which may be of value in medicine; in the near future, many human diseases may be treatable by the consumption of specific plant miRNAs through food. Such findings have been confirmed or partially confirmed by other laboratories or in other experimental settings [24,25,26,27], but have been strongly questioned by other authors in light of a lack of repeatability [16,28,29,30]. Many of these very recent reports, from each side of the controversy, have been reviewed and discussed in the papers by Perge et al. [31] and Lukasik et al. [32]. Clearly, controversy exists as to whether the copy numbers of exogenous sRNAs determined in sequencing studies, especially miRNAs, are high enough to affect the function of a living organism; whether other sRNA species in plant-derived diets might have similar functions; and whether these effects are scalable to the treatment of humans. Scientific as well as technical issues may be the underlying causes of inconsistencies in the results. Contamination of dietary miRNAs with endogenous animal miRNAs may also lead to false positive results. Clearly, the cross-kingdom transfer of plant miRNAs and their therapeutic potential needs to be further investigated and any effects unambiguously confirmed, and any potential risk should also be seriously considered.

## 3. Pathogen miRNAs Regulate Gene Expression in Hosts

A pathogen is any organism, in particular microorganisms, such as bacteria, viruses, protozoa, or fungi, capable of causing disease. Many pathogens of plants and animals often work through protein effectors that are delivered into host cells to disrupt critical cellular functions and then suppress host acquired (adaptive) or innate immunity [33,34]. A number of papers have been published recently demonstrating that sRNAs derived from pathogens can also work as effectors, and subsequently, there has been a research focus on cross-kingdom regulation mediated by exogenous miRNAs in host–pathogen interactions, particularly the role of pathogen-derived miRNAs in regulating defense gene expression in the host [35,36,37,38].

In the challenged plant host, pathogen-derived sRNAs can suppress plant immunity by hijacking the host RNA interference (RNAi) pathway. *Botrytis cinerea* Pers., a fungal pathogen causing gray mold disease, infects more than 200 plant species and annually causes huge economic losses worldwide. Weiberg et al. found that some *B. cinerea* sRNAs (*Bc*-sRNAs) could work as “virulent” effectors in host plant cells [35]. After inoculation of *Arabidopsis* leaves, the plant cells contained a suite of fungal-derived sRNAs. Three sRNAs (*Bc*-siR3.1, *Bc*-siR3.2, and *Bc*-siR5) were found to bind to the *Arabidopsis* Argonaute 1 (AGO1) protein, thereby silencing the plant’s antifungal defense genes [35]. A more recent finding by the same group found that such *Bc*-sRNA effectors were mostly produced by the *B. cinerea* Dicer-like protein 1 (Bc-DCL1) and Bc-DCL2 [36].

Wheat stripe rust, caused by the fungus *Puccinia striiformis* f. sp. *tritici* Westend. (*Pst*), is among the most destructive wheat diseases in the world, being able to greatly reduce or even completely eliminate the yield of a crop [37]. Recently, a study by Wang et al. [38] on a novel miRNA-like RNA (milRNA) in *Pst* found that it acted as a pathogen effector to suppress wheat innate immunity. Using high-throughput sequencing, an sRNA library from *Pst* germ tubes was constructed to comprehensively examine sRNAs in *Pst*. Within the library, Wang et al. identified a unique *Pst* milRNA that was highly expressed (*Pst*-milR1). Using bioinformatic analysis, the target gene for *Pst*-milR1 was identified, not in *Pst*, but in wheat [38]. The wheat gene *SM638*, encoding β-1,3-glucanase, which belongs to the pathogenesis-related 2 (*PR2*) defense gene class in wheat, was predicted to be targeted by *Pst*-milR1 at its 3′ UTR. Subsequently, these authors carried out cotransformation studies and 5′ rapid amplification of the cDNA ends (5′ RACE) by PCR in tobacco leaves and confirmed that *SM638* was effectively targeted by *Pst*-milR1 [38].

To explore the roles of *SM638* and *Pst*-milR1 in pathogenicity, Wang et al. revealed, by utilizing the virus-induced gene silencing (VIGS) approach, that *SM638* could improve *Pst* resistance in an incompatible interaction, and demonstrated, using qRT-PCR and the host-induced gene silencing (HIGS) system, that *SM638* was negatively regulated by *Pst*-milR1 in a wheat–*Pst*-compatible interaction [38]. In addition, the study also showed that *Pst*-milR1 might be a Dicer-dependent sRNA, and that the *Pst* RNase III Dicer-like protein (*PsDCL*) gene might contribute to *Pst* virulence by producing *Pst*-milRNAs [38]. Taking the findings together, the study postulated that *Pst*-milR1, a Dicer-dependent sRNA, was an important pathogenicity factor of *Pst*, which impaired wheat resistance to *Pst* by negatively regulating the wheat *PR2* gene *SM638*.

In animal hosts, expression of pathogen-derived miRNAs during latency may allow for manipulation of host signaling pathways by nonimmunogenic molecules, which can help the latently infected cell to remain primed for reactivation. Human cytomegalovirus (HCMV) is a factor strongly associated with morbidity and mortality in transplant recipients, resulting in hearing loss and mental retardation when acquired congenitally. Previous studies revealed that HCMV caused the induction of antiviral proinflammatory cytokines by inducing host nuclear factor kappa-light-chain-enhancer of activated B cells (NF-κB) signaling early in infection, with a subsequent reduction in the level of these cytokines late in infection [39]. Hancock et al. reported that several viruses have developed mechanisms to block the antiviral effects of these cytokines [40]. These authors showed that two HCMV miRNAs, miR-US5-1 and miR-UL112-3p, targeted the inhibitor of κB (IκB) kinase (IKK) complex components IKKα and IKKβ to limit production of proinflammatory cytokines in response to interleukin 1β (IL-1β) and tumor necrosis factor alpha (TNF-α) [40]. Transfection of miR-UL112-3p and miR-US5-1 mimics reduced the levels of endogenous IKKα and IKKβ protein and achieved site-directed mutagenesis of the 3′ UTRs identified. Combining bioinformatics analysis with dual luciferase reporters, each miRNA was identified to the target 3′ UTRs of IKKα and IKKβ [40]. Subsequently, infection with mutant HCMV lacking the two miRNAs induced increased IKKα and IKKβ protein levels, demonstrating an impaired ability to control NF-κB signaling at late stages of lytic infection, and increased production of proinflammatory cytokines compared to the wild-type virus in cell types relevant to HCMV infection in vivo. These results demonstrated that HCMV-miR-US5-1 and HCMV-miR-UL112-3p specifically downregulated IKKα and IKKβ signaling factors necessary to transmit NF-κB signaling and subsequent production of IL-6, Chemokine (C-C motif) ligand 5 (CCL5), and tumor necrosis factor alpha (TNF-α) [40]. The study indicated that the mechanism by which HCMV miRNAs are expressed late in the infection cycle downregulates proinflammatory cytokine production to create a cellular proviral environment which is critical to viral survival in the host.

Apart from pathogen-derived miRNAs, the parasitic plants dodders (*Cuscuta campestris* Yunck.) can transfer their miRNAs to infected *Arabidopsis thaliana* (L.) Heynh. hosts, which may increase the virulence of the parasite [41]. Taking advantage of sRNA sequencing technology, Axtell and colleagues performed sRNA sequencing of three tissues: the host stem, the interface (that is, the *C. campestris* haustorium and the *A. thaliana* stem), and the parasite stem and compared the small noncoding RNA profiles from the different tissues. The authors found that expression of 76 dodder sRNAs was upregulated at the interface, 42 of which were miRNAs [41]. Furthermore, bioinformatic analysis, 5′-RLM-RACE, qRT-PCR, and mutant experiments indicated that six *Arabidopsis* genes expressed at the interface were plausible targets for these dodder miRNAs [41]. Previous reports revealed that these *Arabidopsis* genes are associated with pathogen-induced signaling (*AFB2*, *AFB3*, *BIK1*, and *TIR1*) and sugar content in detached leaves (*SEOR1*) [42,43,44,45,46]. The authors repeated the sRNA sequencing screen with parasite-infected tobacco (*Nicotiana benthamiana* Domin) and obtained similar results [41]. This work elegantly revealed the induction of parasite-derived miRNAs as a mechanism of cross-kingdom regulation of mRNA expression in multiple hosts. Because some miRNAs are involved in plant immunity against pathogens and insects [3,47], it must be determined whether parasite-derived miRNAs travel to the interfaces between parasitic plants and host plants, altering host immunity against pathogens and pests; and whether root-parasitic plants, such as *Striga* and *Orobanche*, use similar miRNA-based weapons in the establishment and maintenance of plant-plant parasitism [48,49].

## 4. Host miRNAs Regulate Gene Expression in Pathogens

Another model of miRNA-mediated cross-kingdom regulation in pathogen–host interactions is one where specific host-derived miRNAs downregulate expression of the genes essential for virulence in a pathogen, resulting in disease resistance.

Cotton wilt disease, caused by the fungus *Verticillium dahliae* Kleb., is one of the most important diseases of cotton, causing serious reductions in yield and negatively impacting on fiber quality, and it is a difficult disease to prevent or to control effectively [50]. A comprehensive study of the role of RNA silencing in *V. dahliae* pathogenesis has been undertaken [51]. Using deep-sequencing technology and northern blot analysis, Zhang et al. found that high concentrations of two specific cotton plant miRNAs (miR159 and miR166) were exported into the fungal hyphae after infection [51]. Combining computational prediction with 5′ RACE, these authors detected specific cleavages at the predicted binding sites of miR159 and miR166 to isotrichodermin C-15 hydroxylase (*HiC-15*) and Ca^2+^-dependent cysteine protease (*Clp-1*) mRNAs, respectively. The specific targeting of the fungal mRNAs by plant miRNAs was identified subsequently by transiently expressed miRNA-resistant *HiC-15* and *Clp-1* (*HiC-15m* and *Clp-1m*) in tobacco plants and *V. dahliae* [51]. Next, *V. dahliae HiC-15* and *Clp-1* mutant experiments confirmed that both fungal genes were essential for fungal virulence, and that they were specifically targeted by the miRNAs exported from the infected cotton plants to achieve silencing and hence to confer resistance to the fungal pathogen [51]. Subsequently, the role of host miRNAs in specific gene silencing and the consequent reduction in virulence of the fungal pathogen was further verified by comparing *V. dahliae* infection in wild-type *Arabidopsis* plants and in the short-tandem target mimic (STTM) *Arabidopsis* line STTM166, in which miR166 had been degraded [51]. Taken together, this paper presented a description of a novel plant defense strategy against a pathogen by specific silencing of pathogen virulence genes by miRNAs exported from the challenged host plants to the fungal pathogen.

Human papillomavirus (HPV type 16) is regarded as a “high-risk” factor because chronic infection with this virus has been associated with cancers of the cervix, the oropharynx, and head and neck cancer (HNC) [52]. Numerous studies have shown that some host miRNAs are induced during virus infection and participate in the regulation of the innate immunity antiviral response [53,54,55,56]. To investigate the role of host miRNAs in HPV-mediated oncogenesis, Sannigrahi et al. identified a *Homo sapiens* miRNA (Hsa-miR-139-3p) with putative binding sequences on HPV-16 mRNA, using in silico tools and the luciferase reporter assay [57]. These authors found that expression of Hsa-miR-139-3p was strikingly downregulated as a result of increased DNA methylation of the Hsa-miR-139-3p-harboring gene *PDE2A* at its promoter/CpG islands in HPV-16-positive tissues and cell lines [57]. Overexpression and inhibition studies were carried out to establish the role of miRNAs in regulating oncogenic pathways. The results showed that Hsa-miR-139-3p could target high-risk HPV-16 oncogenic proteins and major tumor-suppressor proteins, resulting in inhibition of the growth of HNC and cervical cancer cells. Furthermore, a greater sensitivity to chemotherapeutic drugs (cisplatin and 5-fluorouracil) was observed in Hsa-miR-139-3p-overexpressed HPV-16-positive cells [57]. This paper showed that HPV-16-mediated downregulation of Hsa-miR-139-3p may promote oncogenesis in HNC and cervical cancer.

## 5. Conclusions and Future Prospects

**Figure 1:**
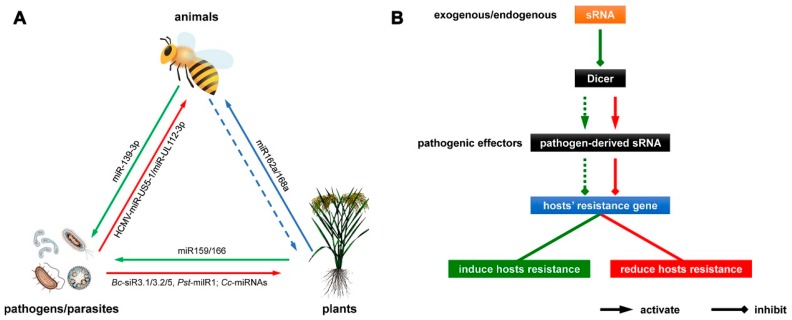
(**A**) The model of miRNA-mediated cross-kingdom regulation. The blue arrow indicates that plant miRNAs (e.g., miR162a and miR168a) regulate gene expression in animals. The blue dotted arrow indicates that no relevant experiment has been reported up to this time. The red and green arrows stand for the model of miRNA-mediated cross-kingdom regulation in pathogen/parasite–host interactions, which indicate that pathogen/parasite miRNAs (e.g., *Bc*-siR3.1/3.2/5, *Pst*-milR1, Cc-miRNAs and HCMV-miR-US5-1/miR-UL112-3p) regulate gene expression in hosts, and host miRNAs (e.g., miR159/166 and miR-139-3p) regulate gene expression in pathogens, respectively; (**B**) New insights into the diagnosis and control of pathogenic diseases. Lines of the same color stand for location on the same pathway. Dotted lines stand for reductions in the positive or negative effects.

The cross-kingdom regulation mediated by exogenous miRNAs means that the miRNAs endogenous to one species may impact the biological processes of another, distantly-related species. Ever since scientists found that miRNAs were not unstable, but played a novel role in interspecies communication, the studies of miRNA-mediated cross-kingdom regulation has focused on two areas: firstly, the regulation by plant miRNAs of gene expression in animals, and secondly, the miRNA-mediated cross-kingdom regulation in pathogen–host interactions (Figure 1B). The former research area usually indicates that plant miRNAs can regulate the expression of target genes in animals and contribute to phenotypic differentiation or to the function of tissues/organs. The latter research area generally includes two research branches, one branch whereby pathogen-derived miRNAs regulate host gene expression to suppress host disease resistance, while the other branch involves host-derived miRNAs in the regulation of pathogen gene expression to confer disease resistance (Figure 1B).

These findings provide new evidence for the existence of cross-kingdom regulation mediated by exogenous miRNAs. Nevertheless, some questions remain unanswered. The three prime issues are as follows: Firstly, a clear molecular mechanism by which miRNAs endogenous to one species can be incorporated into another, distantly related species needs to be confirmed. Hitherto, two different mechanisms of dsRNA uptake have been described. One involves the use of the systemic RNA interference deficient (SID) transmembrane channel-mediated proteins, which were reported in a nematode (*Caenorhabditis elegans* Maupas), a planarian (*Schmidtea mediterranea* Benazzi et al.), and the Colorado potato beetle (*Leptinotarsa decemlineata* Say) [58]. Some SID proteins (SID-1/SID-2) are necessary in the systemic RNAi response. For SID-1, homologous genes have been found in many, but not all, insects, whereas for SID-2, no insect homologs have been reported to date [59]. The other dsRNA uptake mechanism is via microvesicle (MV) compartments, the intracellular carriers of endogenously originating miRNAs, including shedding vesicles (SVs), exosomes, and apoptotic bodies, which are derived from the cell surface, the endosomal membrane, and the plasma membrane, respectively [60,61]. These vesicles have reported to protect miRNAs from degradation by RNases [62,63]. In addition to animal body fluids, exosome-like nanoparticles containing proteins, lipids, and RNAs have also been found in many plant sources, such as ginger, carrot, watermelon, grapes, olives, melon seeds, and coconut [25,64,65,66,67]. Published studies have suggested that exosome-like nanoparticles derived from plants may mediate interspecies communication and induce the expression of certain human genes [64]. Whether one or both of these pathways of dsRNA uptake are present and play equivalent roles in sRNA transport needs further research.

Secondly, the strategy needs to be identified by which the appropriate dosage of miRNAs from one species is delivered to the other species in order to modulate the relevant biological processes. Ke-Gan Zhu et al. used the same dose of plant miRNAs as exists in natural beebread to feed honey bee larvae, producing results similar to those seen in nature [17]. Even less is known as to whether the organisms taking up exogenous miRNA possess an amplification pathway, such as that of the transitive RNAi found in *C. elegans*. Transitive RNAi is a model in which mRNA targeted by RNAi functions as a template for de novo synthesis of new sRNA. In this model, hosts used the available dosage of exogenous miRNAs to produce considerable amounts of secondary sRNAs to ensure a robust RNAi response [68].

The third open question is how miRNAs endogenous to one species are loaded onto Argonaute proteins of the other, distantly-related species to produce a functional miRNA form. It is generally known that miRNAs derived from different species regulate the expression of their targeting gene in different ways. Specifically, plant miRNAs usually have near-perfect pairing with the coding region of their targets, causing target mRNA to break down, while animal miRNAs possess a sequence incompletely complementary to the 3′ UTR of their targets by binding to as little as 6–8 nucleotides (the seed region) at the 5′ end of the miRNA, blocking translation [69]. In the example where a plant miRNA in larval food regulated honey bee caste development, the way in which miR162a reduced *amTOR* expression in vivo resembled that of a plant miRNA, whereas miR162a showed incomplete complementarity with the *amTOR* sequence and showed a G:U wobble in the seed region, resembling the regulatory action of an animal miRNA [17]. How exogenous miRNAs are incorporated into different species, forming miRNA–Argonaute complexes, has still to be satisfactorily explained.

Coevolution influences the structure and function of ecological communities as well as the dynamics of infectious diseases [70]. Recently, it has been found that miRNA-mediated cross-kingdom regulation could be involved in pathogen–host interactions, and this new model of horizontal miRNA transfer might blaze a new trail to dissect the molecular mechanisms underlying cross-kingdom interaction and coevolution. Zhang et al. reported that cotton miRNAs miR159 and miR166 negatively regulated the expression of *HiC-15* and *Clp-1*, respectively, these target genes being essential for virulence, so that the miRNAs conferred disease resistance to the cotton plant [51]. The novel plant defense strategy against a pathogen, mediated by cross-kingdom regulation of miRNA, was also found in tomato plants [51]. Meanwhile, Zhang et al. remarked that the pathogen sequences targeted respectively by miR159 and miR166 were highly conserved among different strains of *V. dahliae*, especially with respect to the miRNA-binding regions [51]. These findings indicated that pathogens might have preserved or evolved this miRNA-dependent regulation to prevent host hypersensitive responses and to keep the host tissues alive during the biotrophic phase of the infection. It is also possible that host miRNAs do not target modulation of the pathogen virulence genes, in view of coevolution during a long-term specific host–pathogen interaction.

These studies, which focused on the cross-kingdom regulation mediated by exogenous miRNAs in host–pathogen interactions, described a novel host defense strategy against pathogenic infection and a novel virulence strategy to overcome host defenses (Figure 1B). These findings reflected, in fact, the central role of miRNAs in the regulation of gene expression and its implications for miRNA-specific aberrant expression in the pathogenesis of cancer, cardiac, metabolic, neurologic, and immune-related diseases, as well as in many plant diseases [71], providing new insights into the diagnosis, prognosis, and treatment of human and crop diseases. Taking the plant–pathogen example, pathogens deliver sRNAs into plant cells to silence host innate immunity genes. Such sRNA effectors of pathogens are mostly produced by Dicer-like proteins (DCLs) of pathogens. Wang et al. reported that expressing internal and external sRNAs that target DCLs of pathogens in *Arabidopsis* and tomato silences the *DCL* genes of pathogens and attenuates pathogenicity of and colonization by pathogens, exemplifying bidirectional cross-kingdom RNAi and sRNA transfer between plants and pathogens [36]. This strategy could potentially be adapted to simultaneously control multiple diseases caused by a range of pathogen sRNAs (Figure 1B). Another preventative disease-resistance strategy occurs when the infected hosts export specific miRNAs to induce specific cross-kingdom silencing of pathogen virulence genes and hence induce resistance. The molecular mechanisms of miRNA-mediated cross-kingdom regulation in host plant–pathogen interactions represent potentially a new generation of environmentally-friendly crop protection chemicals and could be effective in crop improvement and the treatment of crop diseases in the future.

Herbal medicine, in which plant extracts are by far the most common elements used, accounts for the majority of treatments in traditional Chinese medicine (TCM) [72]. Exogenous plant miRNAs can be transferred to animals, and hence contribute to some important examples of phenotypic regulation in animals, indicating that plant miRNAs contained in herbal medicines might also act as a type of active ingredient. Radix glycyrrhizae, the root of Chinese liquorice (*Glycyrrhiza uralensis* Fisch.), is one of the 50 fundamental herbs used in TCM, and has multiple pharmacological properties such as anti-inflammatory, antiviral, antimicrobial, antioxidative, antidiabetic, antiasthma, and anticancer activities as well as immunomodulatory, gastroprotective, hepatoprotective, neuroprotective, and cardioprotective effects [73]. Studies showed that there were abundant miRNAs in the decoction of dried liquorice [74]. Researchers treated peripheral blood mononuclear cells (PBMC) isolated from healthy volunteers with miRNAs extracted from a *G. uralensis* decoction as well as synthesized miRNA mimics, and found that glycyrrhiza miRNA could significantly regulate PBMC by inhibiting the expression of genes involved in T-cell differentiation, inflammation, and apoptosis [75]. The mechanisms of miRNA-mediated cross-kingdom regulation could bring about new strategies in TCM, allowing a comprehensive study of the pharmacology of TCM, improving the development of TCMs and generating new drugs.

Whether it is transferred between species and mediates cross-kingdom regulation by integrating into a specific target-gene-mediated regulatory pathway or a complex regulatory network, miRNA has huge potential to work as a major factor to influence biological functions in the host. There is no doubt that in the future, the identification, confirmation, and analysis of more miRNA molecules, as well as a greater understanding of miRNA-mediated cross-kingdom regulation, will result in the application of miRNAs for the diagnosis, prognosis, and treatment of human diseases; crop improvement by increasing disease resistance; and the pharmacological study of traditional Chinese medicines.
